# Results of a person-centered maternal health quality improvement intervention in Uttar Pradesh, India

**DOI:** 10.1371/journal.pone.0242909

**Published:** 2020-12-11

**Authors:** Dominic Montagu, Katie Giessler, Michelle Kao Nakphong, Kali Prasad Roy, Ananta Basudev Sahu, Kovid Sharma, Cathy Green, May Sudhinaraset

**Affiliations:** 1 Department of Epidemiology and Biostatistics, University of California, San Francisco, San Francisco, CA, United States of America; 2 School of Public Health, University of California, Los Angeles, Los Angeles, CA, United States of America; 3 Population Services International, New Delhi, India; 4 Independent Consultant, Cape Town, South Africa; BITS Pilani, INDIA

## Abstract

**Background:**

Poor patient experiences during delivery in Uttar Pradesh, India is a common problem. It delays presentation at facilities after the onset of labor and contributes to poor maternal health outcomes. Patient-centered maternity care (PCMC) is recognized by the World Health Organization as critical to overall quality. Changing PCMC requires changing the process of care, and is therefore especially challenging.

**Methods:**

We used a matched case-control design to evaluate a quality improvement process directed at PCMC and based on widely established team-based methods used in many OECD countries. The intervention was introduced into three government facilities and teams supported to brainstorm and test improvements over 12 months. Progress was measured through pre-post interviews with new mothers, scored using a validated PCMC scale. Analysis included chi-squared and difference-in-difference tests.

**Findings:**

On a scale to 100, the PCMC score of the intervention group increased 22.9 points compared to controls. Deliveries attended by midwives, dais, ASHAs or non-skilled providers resulted in significantly higher PCMC scores than those attended to by nurses or doctors. The intervention was associated with one additional visit from a doctor and over two additional visits from nurses per day, compared to the control group.

**Interpretation:**

This study has demonstrated the effectiveness of a team-based quality improvement intervention to ameliorate women’s childbirth experiences. These improvements were locally designed and led, and offer a model for potential replication.

## Background

The Indian state of Uttar Pradesh has seen a substantial decrease in its maternal mortality ratio (MMR), with a reduction from 285 per 100,000 live births in 2011–13 [1] to 201 deaths per 100,000 live births in 2014–16 [2]. Although this is a reduction of nearly 30%, Uttar Pradesh’s MMR remains one of the highest in India.

An ongoing contributor to high maternal mortality rates across low-resource settings is poor quality of facility-based care [3]. Poor quality care, whether clinical or interpersonal, often deters women from accessing healthcare for both urgent current needs and in the future, regardless of necessity [4–6]. Interventions to improve the quality of facility-based care are likely to contribute to improved utilization of formalized reproductive and intrapartum healthcare by women, and in this way reduce avoidable morbidity and mortality for this population [7]. The importance of person-centered maternity care (PCMC) and client experience is appreciated as a central component to overall maternal health quality [8].

### Benefits of person-centered maternity care

PCMC includes multiple dimensions of care that patients experience in a facility and the environment in which a woman seeks care, including interpersonal interactions, freedom from coercion and abuse, informed and consented care, and provision of respectful care [6, 9]. Better PCMC is associated with higher patient satisfaction, earlier presentation for care, improved adherence to post-care treatment, and lower health care costs overall [10, 11]. When women trust that the care they will receive in a facility will be humane, collaborative, safe and respectful, they are more likely to access care in general and at an earlier point, as well as follow provider guidance for treatment, thus potentially reducing maternal complications and mortality in the longer term. In Kenya, better PCMC has been shown to be associated with lower rates of newborn complications and higher willingness to return to a health facility in the future [Sudhinaraset et al. forthcoming].

### Person-centered maternity care in Uttar Pradesh

Experiences of person-centered care in Uttar Pradesh have been documented in a number of settings, all studies concluding that significant quality shortcomings are commonplace, particularly within the public health facilities which serve 45% of women giving birth [12, 13]. Verbal and physical abuse affect more than a third of all women in Uttar Pradesh [14]. Slapping, shouting, and other forms of abuse during delivery are reported by more than a half of all women, and less egregious forms of mistreatment or disrespect are ubiquitous [6, 13]. In Uttar Pradesh, mistreatment is associated with increased odds of delivery and postpartum complications in [10]. Poor person-centered care affects women’s avoidance of facilities, leading to significant increased health risks from delays in seeking care after the onset of labor [4, 15, 16]. Once at a facility, poor person-centered care can delay the recognition of complications, the decision to treat or refer, and limit the amount of information that is shared with a receiving facility, thereby making referrals more difficult and generating higher risk of complication for the woman being referred [17]. Poor person-centered care during delivery is predicted to have lasting effects on mothers’ decisions regarding returning for post-partum check-ups, well-baby care, and on health seeking decisions for future births both by the mother and by other women in her community [4, 18].

Problems of poor person-centered care are more prevalent in larger facilities, and are understood to be driven by the process of care provision, rather than infrastructure of the facility where care occurs [19]. Because of this, improvements require changing the norms of treatment practice, and are not easily or rapidly effected. Changing person-centered maternity care is, nonetheless, a priority for the government, and is core to the guidelines of the new national labor room quality improvement initiative, LaQshya [20]. Identifying effective ways to improve person centered care for maternal health services has the potential to inform and complement national healthcare strategies to improve quality of care overall. We adapted and applied a team-based quality improvement methods used by hospitals in many OECD countries [21].

## Methods

In collaboration with the National Health Mission (NHM) of Uttar Pradesh, we conducted a matched case-control quality improvement (QI) intervention to improve the PCMC provided to women delivery in government facilities as a first step to identifying interventions which might be scaled across the state. All nine study sites were initially identified by the NHM through their participation in the BetterBirth study [22], a large-scale, clinical quality improvement research intervention recently concluded. This was done with the intent to assure that all facilities began with an established acceptable level of clinical care provision; and to make work with facilities easier, as all had experience with external quality improvement partners. Leadership capacity to support a quality improvement intervention focused on PCMC additionally informed site selection. A maximum 4-hour travel time from the study offices in Lucknow, and averaging more than 100 deliveries per month were a criteria for selection to ensure enough participants to assess changes in a composite PCMC score from baseline to endline, both during the QI intervention and during the evaluation of the intervention. We limited ourselves to two districts to reduce between-site travel. Sites were selected from low- to mid-level facilities: either a Primary Health Center (PHC) or a Community Health Center (CHC). Nine facilities in Unnao and Kanpur Districts met our criteria: three PHCs and six CHCs. Three facilities (two CHCs and one PHC) were randomly selected for the intervention and three additional facilities matched as controls based on delivery volume and level of care. (Table 1). The final three facilities were retained as controls for a subsequent stage of the study.

**Table 1:** Demographic characteristics of study participants across intervention vs. control facilities from baseline to endline.

	Baseline					Endline			
	Interven-tion	Control	Total	P-value	Interven-tion		Control	Total	P-value
**Total number in group**	285	285	570		300		300	600	
**Age**	** **	** **	** **	** **	** **		** **	** **	** **
15–19 years	(2.1%)	(2.1%)	(2.1%)	0.972	6		8	14	0.736
20–29 years	(82.8%)	(83.5%)	(83.2%)		(83.7%)		(84.7%)	(84.2%)	
30–40 years	(15.1%)	(14.4%)	(14.7%)		(14.3%)		(12.7%)	(13.5%)	
**Number of births**	** **	** **	** **	** **	** **		** **	** **	** **
1	(36.8%)	(31.6%)	(34.2%)	0.159	(40.7%)		(41.3%)	(41.0%)	0.089
2	(33.3%)	(34.7%)	(34.0%)		(26.3%)		(32.7%)	(29.5%)	
3	(13.7%)	(20.0%)	(16.8%)		(19.0%)		(17.7%)	(18.3%)	
4 or more	(16.1%)	(13.7%)	(14.9%)		(14.0%)		(8.3%)	(11.2%)	
**Employed**	** **	** **	** **	** **	** **		** **	** **	** **
Yes	(2.5%)	(6.3%)	(4.4%)	0.024	(2.0%)		(1.7%)	(1.8%)	0.761
No	(97.5%)	(93.7%)	(95.6%)		(98.0%)		(98.3%)	(98.2%)	
**Wealth Quintiles**	** **	** **	** **	** **	** **		** **	** **	** **
1	(40.7%)	(20.0%)	(30.4%)	0.000	(13.3%)		(7.0%)	(10.2%)	0.022
2	(21.4%)	(21.8%)	(21.6%)		(18.7%)		(20.0%)	(19.3%)	
3	(12.6%)	(21.8%)	(17.2%)		(24.3%)		(20.0%)	(22.2%)	
4	(14.4%)	(16.8%)	(15.6%)		(23.7%)		(25.0%)	(24.3%)	
5	(10.9%)	(19.6%)	(15.3%)		(20.0%)		(28.0%)	(24.0%)	
**Religion**	** **	** **	** **	** **	** **		** **	** **	** **
Hindu	(91.9%)	(96.1%)	(94.0%)	0.034	(91.7%)		(94.7%)	(93.2%)	0.203
Muslim	(8.1%)	(3.9%)	(6.0%)		(8.3%)		(5.0%)	(6.7%)	
None	(0.0%)	(0.0%)	(0.0%)		(0.0%)		(0.3%)	(0.2%)	
**Caste**	** **	** **	** **	** **	** **		** **	** **	** **
Scheduled Caste	(51.6%)	(43.5%)	(47.5%)	0.001	(50.3%)		(42.7%)	(46.5%)	0.023
Scheduled Tribe	(2.5%)	(1.4%)	(1.9%)		(1.7%)		(0.3%)	(1.0%)	
General Caste	(26.3%)	(20.7%)	(23.5%)		(9.7%)		(16.0%)	(12.8%)	
OBC	(19.6%)	(34.4%)	(27.0%)		(38.3%)		(41.0%)	(39.7%)	
**Literate**	** **	** **	** **	** **	** **		** **	** **	** **
No	(30.9%)	(19.6%)	(25.3%)	0.002	(20.0%)		(11.3%)	(15.7%)	0.003
Yes	(69.1%)	(80.4%)	(74.7%)		(80.0%)		(88.7%)	(84.3%)	
**Highest grade/class completed**	** **	** **	** **	** **	** **		** **	** **	** **
No education	(30.9%)	(19.6%)	(25.3%)	0.000	(20.3%)		(13.0%)	(16.7%)	0.000
Primary or post-primary	(41.1%)	(35.8%)	(38.4%)		(50.3%)		(37.3%)	(43.8%)	
Secondary or higher	(28.1%)	(44.6%)	(36.3%)		(29.3%)		(49.7%)	(39.5%)	
**Number of ANC visits**	** **	** **	** **	** **	** **		** **	** **	** **
Less than 4	(67.7%)	(54.7%)	(61.2%)	0.000	(32.3%)		(33.0%)	(32.7%)	0.007
4 or 5	(22.5%)	(43.9%)	(33.2%)		(22.0%)		(32.0%)	(27.0%)	
6 plus	(9.8%)	(1.4%)	(5.6%)		(45.7%)		(35.0%)	(40.3%)	
**Perceived distance to facility**	** **	** **	** **	** **	** **		** **	** **	** **
Very less	(19.0%)	(10.5%)	(14.8%)	0.003	(48.0%)		(39.0%)	(43.5%)	0.024
A little long	(58.6%)	(68.4%)	(63.5%)		(37.7%)		(43.7%)	(40.7%)	
Long	(16.8%)	(19.0%)	(17.9%)		(11.0%)		(16.0%)	(13.5%)	
Very Long	(5.61%)	(2.1%)	(3.9%)		(3.3%)		(1.3%)	(2.3%)	
**Problems during pregnancy**	** **	** **	** **	** **	** **		** **	** **	** **
No	(29.5%)	(70.5%)	(50.0%)	0.000	(70.7%)		(61.3%)	(66.0%)	0.016
Yes	(70.5%)	(29.5%)	(50.0%)		(29.3%)		(38.7%)	(34.0%)	
**Facility Type**	** **	** **	** **						
Government hospital	(32.3%)	(32.6%)	(32.5%)	0.929	(66.7%)		(66.7%)	(66.7%)	1.000
Gov't Health Center	(67.7%)	(67.4%)	(67.5%)		(33.3%)		(33.3%)	(33.3%)	
**Delivery Assistant**	** **	** **	** **	** **	** **		** **	** **	** **
Nurse/Doctor	(12.3%)	(7.0%)	(9.6%)	0.000	(27.7%)		(37.3%)	(32.5%)	0.000
Midwife/Dai	(10.9%)	(16.5%)	(13.7%)		(72.0%)		(51.0%)	(61.5%)	
ASHA/Angawali	(4.6%)	(30.9%)	(17.7%)		(0.3%)		(10.7%)	(5.5%)	
Other/Non-skilled attendant	(72.3%)	(45.6%)	(58.9%)		(0.0%)		(1.0%)	(0.5%)	
**Gender of delivery assistant**	** **	** **	** **	** **	** **		** **	** **	** **
Male	(0.0%)	(0.0%)	(0.0%)	---	(0.3%)		(0.3%)	(0.3%)	1.000
Female	(100.0%)	(100.0%)	(100.0%)		(99.7%)		(99.7%)	(99.7%)	

The facilities were all rural or semi-rural in location and in districts selected to be broadly reflective of the average socio-economic distribution of Uttar Pradesh state. The initial approach to facilities was made to the Medical Superintendents at both intervention and control facilities. Most, if not all, acknowledged that patient interaction was a weak area, and baseline interviews demonstrated this for the topics we focused on. All facilities recruited agreed to participate in the study.

### Intervention

Baseline data was used to identify PCMC indicators where intervention facilities were performing poorly. In each of the three intervention facilities, a Quality Improvement Team (QI Team) was established by the Medical Officer-In Charge of each intervention facility, with guidance from external QI experts. QI Teams included staff most actively involved in maternity care, such as staff nurses, Lady Medical Officers, Dais (traditional birth attendants recruited to work in the facility), and cleaners.

All QI Teams participated in an “Improvement Collaborative”, a model adapted from the Institute for Healthcare Improvement’s Breakthrough Series [4, 23]. All intervention sites worked to improve the same PCMC indicators within their respective sites. The Improvement Collaborative extended across 9 months and was comprised of quarterly workshops, during which QI Teams came together to identify possible reasons for their poorer performance on specific PCMC indicators, agree upon facility and collaborative-based aims for improvement, and identify possible changes they could make that may result in improvement on the specified PCMC indicators. The 3-month activity periods between workshops gave QI Teams an opportunity to experiment with behavior and process changes that might improve PCMC performance.

The Model for Improvement (MFI) [24] guided the QI activity. The MFI encourages the articulation of a clear aim, on-going measurement to establish if there is improvement over time, and the identification of changes believed to contribute to improvement based on systematic problem analysis. Ideas are tested individually using a structured experimental approach known as the Plan-Do-Study-Act cycle to ascertain if changes have contributed to any improvements in key outcomes. In all intervention sites, teams worked on five common Person-Centered Care topics: calling patients by their name; improving cleanliness of the toilets, washrooms and post-natal ward; explaining the purpose of giving medication or tests undertaken; and ensuring patients were covered with a blanket of cloth while in the labour area. Beyond this, each facility worked to improve one of three additional topics: giving pain medication when the patient felt they needed it, accompanying the woman to the toilet, or accommodating to the patient’s preferred position during labour and delivery.

To change practices related to each of these topics the staff in each facility generated and tested ideas. Examples of ideas for improvement included: reminders of the importance of targeted change practices through posted visual notes; providing weekly feedback on any changes in performance; identifying outliers and encouraging compliant behavior through a senior team member; linking new behaviors with standard routines such as coming on duty or entering the post-natal care ward; practicing new behaviors with peers; ensuring near-to-patient storage of essential inputs (e.g. water and pain medication; blankets to assure privacy); or developing standard script for explaining medicines. Changes were initially introduced on a very small scale and if the change showed promise to generate improvement, it was adapted and re-tested until the benefits achieved ‘face-validity’. For this, QI Team members conducted exit interviews with women who had delivered within the past 7 days and then made adjustments to interventions as a result of these interviews.

QI Teams at each intervention facility received weekly support visits from a QI specialist to assist in interpreting and plotting exit interview data, assess the efficacy of change strategies, and provide mentorship on implementation of PDSA cycles. QI Team meetings lasted one to two hours and were conducted during normal working hours at times identified as most convenient by team members so as to avoid interruption of their clinical duties.

### Data collection

The baseline survey was conducted between September 2016 and March 2017. The endline was conducted in two waves between May and December 2018. In total, 570 women were surveyed at baseline and 600 at endline from three intervention and three control facilities. Inclusion criteria were women aged 18–49 years who had recently delivered at the health facility in the last seven days and who were willing and consented to participate. Women who had delivered outside of a participating health facility, were sick at the time of recruitment, were less than 18, or who refused to participate following a short explanation about the study purpose were excluded from participating in the survey.

Surveys were conducted using a pre-tested, structured questionnaire. Local investigators were recruited and trained to conduct informed consent and administer the survey via a web-based application. Quality checks (skip patterns, relevance and constraints) were developed in the application and surveys reviewed by the local Research Manager nightly to ensure quality and accuracy. Investigators provided a paper copy of the consent form for reference and verbally read the entire form to each potential study participant prior to enrollment. Women who agreed to participate in the study were then verbally consented by the investigator and their consent noted within the web-based survey application prior to starting the survey. All surveys were conducted in person at the health facility in the most private setting available and each survey took approximately 45 minutes.

### Ethics compliance

Human subjects approval for this study was received in both the United States, from the University of California, San Francisco (IRB# 15–18008, ref 176940; 11/09/2016), and in India, from the Public Health Foundation of India (TRC-IEC-276/15; May 2, 2016).

## Outcome variables

### Person-centered maternity care

Person-centered maternity care (PCMC) was assessed using a validated scale that measures care received within three domains: dignity and respect; communication and autonomy; and supportive care. This scale was validated using survey data from women who had delivered in Uttar Pradesh specifically and contains 27 items to measure the woman’s PCMC experience at the facility [25]. Of the original 27 PCMC scale items, 23 were assessed. Data was not collected for items regarding friendliness, being called by name, consent to procedures, and talking about how one felt as these items were not finalized in the scale at the time of the baseline survey. One question differed from the validated scale. For the item about providers’ introduction, we used the question “Did the doctors, nurses or other health care providers introduce themselves to you when they came to see you?” Each item asked about frequency of person-centered experiences or care received and scores on individual items ranged from 0 to 3 (0 “No never”; 1 “Yes, a few times”; 2 “Yes, most of the time”; 3 “Yes, all of the time”). Responses that were recorded as “not applicable” were conservatively recoded to receive the highest score. Total PCMC scores were calculated by summing all items for each participant, ranging from zero to 69 points. Final total PCMC and subdomain scores were scaled to 100-point scales.

### Other outcome variables

We examined the impact of the intervention on other outcomes including clinical quality, delivery complications (yes vs. no), and frequency of doctor and nurse visits while in the maternity ward (number of visits per day. Clinical quality was measured by a clinical quality index constructed from 25 items asked of women regarding procedures received while at the facility such as checking blood pressure, pulse, or vaginal exam, among others (yes vs. no). Responses recorded as “don’t know” were recoded as “no.” Possible scores for the index ranged from zero to 25.

### Other associated variables

We examined factors that may be associated with PCMC and other outcomes including socioeconomic factors, pregnancy characteristics, and provider characteristics. We investigated the distributions of age, parity, employment, wealth, religion, caste, literacy, education, number of antenatal care visits, perceived distance to the facility, pregnancy complications, facility type, as well as type and gender of delivery assistant. Wealth was assessed by a modified EquityTool based on India NFHS4 [Released March 30, 2019], equitytool.org, maintained by Metrics for Management.

## Analysis

We compared the intervention and control groups at baseline and endline via bivariate analyses. Differences between groups at each timepoint were assessed by cross-tabulations, chi-square tests, and t-tests. We examined the impact of the intervention on outcomes using multivariate regression difference-in-differences models. Models included a main effects term for both survey round and treatment group as well as an interaction term between survey and treatment to indicate the difference in groups over time. Models for PCMC, clinical quality index, frequency of doctor and nurse visits, and wait time used ordinary least square regression. Logistic regression was used to evaluate the odds of delivery complications. We tested for homogeneity of variance and used robust standard errors (Eicker-Huber-White) to correct for homoschedasticity and clustering. Final multivariate models adjusted for age, parity, education, wealth, religion, caste, perceived distance to the facility, facility type, delivery provider, ANC visits, and pregnancy complications Stata SE 15.1 was used for all analyses. Because we investigated a primary outcome associated with the intervention treatment, we conducted a planned comparison [26] for which statistical significance was established at an alpha level of 0.05. For additional outcomes, we performed Bonferroni corrections and reported outcomes for which the p-value of the interaction term reached critical significance (*α_critical_* = 0.005).

## Results

Participants at intervention facilities tended to have less wealth, less education, fewer ANC visits, and differed according to caste than those at control facilities (Table 1). At baseline, the intervention group had more Muslim participants, but no significant difference was observed at endline. More intervention facility participants also had pregnancy complications than those at control facilities, but these trends reversed at endline. Though groups differed by delivery provider at both survey rounds, we observed a trend of more deliveries assisted by skilled attendants in both groups over time; the majority of deliveries in both groups were attended by non-skilled assistants at baseline, whereas the majority of endline deliveries were attended by a midwife/Dai. Baseline total PCMC and subdomain scores were higher at intervention facilities compared to controls, but over time, scores at intervention facilities observed increases in PCMC scores, while scores at control facilities decreased (Table 2).

**Table 2:** Baseline-endline all.

	Baseline				Endline			
	Intervention		Control		Intervention		Control	
	Mean	SD	Mean	SD	Mean	SD	Mean	SD
**Total PCMC score**	** **	** **	** **	** **	** **	** **	** **	** **
PCMC total sum (all 27 indicators)	80.94	(8.87)	73.17	(10.38)	97.02	(3.10)	60.91	(11.47)
PCMC total sum (validated scale: 23 items)	80.77	(8.03)	73.63	(9.47)	97.13	(2.91)	63.42	(11.44)
Dignity and Respect domain subtotal	94.41	(9.93)	77.87	(11.22)	98.22	(3.63)	77.42	(15.65)
Communication and Autonomy domain subtotal	78.56	(13.06)	68.25	(17.97)	96.89	(5.37)	40.98	(16.24)
Supportive Care domain subtotal	75.97	(9.63)	75.13	(13.22)	96.78	(3.00)	71.33	(11.57)
**Specific Indicators**								
**Dignity and Respect Domain**								
Treated with respect	2.95	(0.28)	2.88	(0.42)	3	(0.00)	2.14	(0.76)
Visual privacy	2.44	(1.17)	0.81	(1.28)	2.99	(0.17)	1.64	(1.01)
Record confidentiality	2.8	(0.64)	2.01	(1.09)	2.76	(0.51)	2.18	(0.90)
Verbal abuse	2.98	(0.17)	2.98	(0.13)	2.99	(0.11)	2.69	(0.68)
Physical abuse	3	(0.00)	3	(0.00)	3	(0.00)	2.96	(0.27)
**Communication and Autonomy Domain**								
Introduce self	0.36	(0.97)	0.01	(0.19)	2.61	(0.74)	0.09	(0.30)
Involvement in care	2.82	(0.52)	2.55	(0.88)	2.93	(0.25)	1.08	(1.11)
Delivery position choice	2.81	(0.63)	2.8	(0.60)	2.91	(0.36)	1.22	(1.11)
Language	2.62	(0.88)	2.27	(1.02)	2.97	(0.17)	2.45	(0.78)
Explain exams/procedures	2.83	(0.49)	2.42	(0.87)	2.94	(0.24)	0.68	(0.91)
Explain medicines	2.21	(1.19)	1.84	(1.12)	3	(0.06)	1.06	(1.24)
Able to ask questions	2.85	(0.55)	2.45	(0.95)	2.98	(0.15)	2.02	(0.91)
Explain purpose**	2.21	(1.19)	1.84	(1.12)	2.85	(0.51)	0.89	(0.97)
**Supportive Care Domain**								
Time to care	0.06	(0.43)	0.73	(1.29)	2.91	(0.30)	2.44	(0.78)
Labor support	2.89	(0.41)	2.92	(0.39)	3	(0.00)	2.75	(0.57)
Delivery support	2.92	(0.42)	2.91	(0.41)	3	(0.00)	2.72	(0.60)
Attention when need help	2.85	(0.47)	2.73	(0.65)	2.99	(0.10)	1.98	(0.80)
Control pain	2.45	(1.03)	1.48	(1.05)	2.95	(0.22)	1.61	(0.80)
Bribes	1.35	(1.24)	1.82	(1.08)	2.55	(0.60)	2.28	(0.71)
Enough staff	2.77	(0.70)	2.61	(0.76)	2.98	(0.25)	1.87	(0.70)
Took best care	2.86	(0.46)	2.69	(0.66)	2.99	(0.11)	1.81	(0.62)
Trust	2.94	(0.32)	2.77	(0.57)	2.97	(0.16)	2.14	(0.81)
Clean pre-natal care ward**	2.38	(0.98)	2.18	(1.00)	2.89	(0.33)	2.15	(0.86)
Safe	2.93	(0.31)	2.87	(0.39)	2.99	(0.10)	2.26	(0.81)
Asked about pain**	2.55	(0.89)	1.73	(1.14)	2.95	(0.23)	1.22	(0.88)
Helped to the toilet**	2.68	(0.76)	2.71	(0.77)	2.88	(0.48)	1.31	(1.25)
Clean bathroom	1.06	(1.29)	1.25	(1.40)	2.61	(0.51)	1.68	(0.62)

**not included in the validated scale total or domain subtotals.

### Impact of the intervention

**Fig 1:**
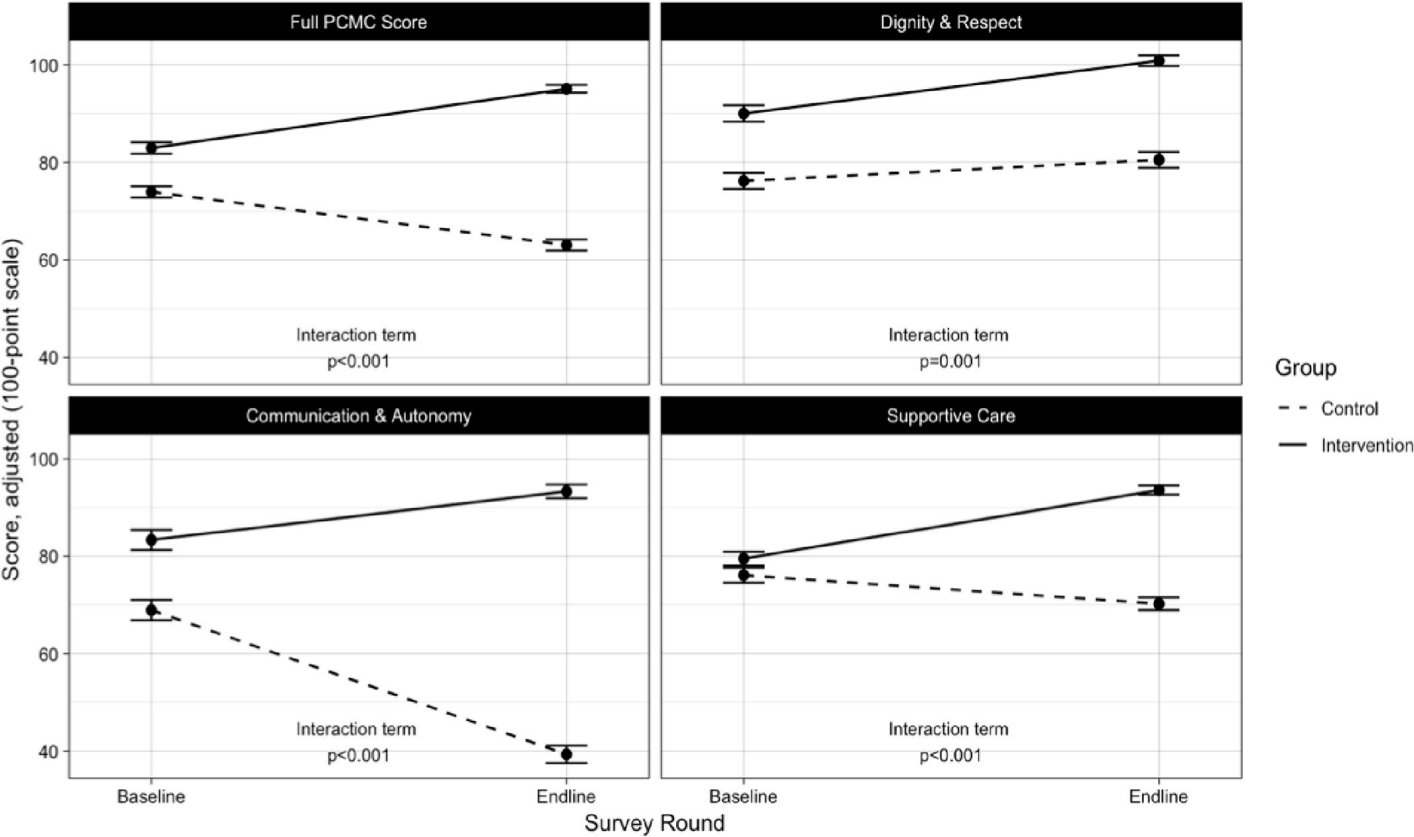
Impact of intervention on total PCMC score and subdomains. *All estimates adjusted for age, parity, education, wealth, caste, facility type, delivery provider, ANC visits, and pregnancy complications. Robust standard errors were used.

Across time, the intervention was associated with positive increases in total PCMC score and all subdomains relative to control facilities (Fig 1). From baseline to endline, the adjusted mean PCMC score of the intervention group increased 22.9 points (95%CI: 20.9, 25.0) compared to controls (Table 3). Regarding other factors, deliveries attended to by midwives, dais, ASHAs or non-skilled providers resulted in significantly higher PCMC scores than those attended to by nurses or doctors (p<0.001). Number of ANC visits was positively correlated with higher PCMC scores while occurrence of pregnancy complications resulted in lower PCMC scores (p<0.001).

**Table 3:** Difference endline-minus-baseline, by intervention/control, for each category.

	Survey Round Endline (reference Baseline)	Treatment Group Intervention (reference Control)	Interaction term
**Full PCMC score (unadjusted)**	** **	** **	** **
Coefficient	-10.21	7.13	26.57
95%CI	(-11.60, -8.82)	(5.73, 8.54)	(24.61, 28.54)
p-value	0.000	0.000	0.000
**Full PCMC score (adjusted)**			
Coefficient	-10.53	9.18	22.93
95%CI	(-12.15, -8.91)	(7.42, 10.93)	(20.85, 25.00)
p-value	0.000	0.000	0.000
**Dignity & Respect (adjusted)**			
Coefficient	4.93	14.14	6.31
95%CI	(2.34, 7.52)	(12.23, 16.06)	(3.83, 8.79)
p-value	0.000	0.000	0.000
**Communication and Autonomy (adjusted)**			
Coefficient	-28.88	14.73	39.42
95%CI	(-31.79, -25.98)	(11.77, 17.69)	(35.97, 42.87)
p-value	0.000	0.000	0.000
**Supportive Care (adjusted)**			
Coefficient	-5.88	3.39	19.99
95%CI	(-7.97, -3.80)	(1.18, 5.60)	(17.36, 22.61)
p-value	0.000	0.003	0.000

The intervention was also associated with a 4.2-point (95%CI: 3.3, 5.2) increase in clinical quality index over time compared to the control group (Table 4). Across time, the odds of delivery complications at intervention facilities were 97% lower (95%CI: 94%, 99%) than at control facilities. Between baseline and endline, the intervention was also associated with an additional visit from a doctor and over two additional visits from nurses per day, compared to the control group.

**Table 4:** Impact of intervention on other health outcomes.

	Baseline			Endline		
	Intervention	Control	Total	Intervention	Control	Total
**Total number in group**	285	285	570	300	300	600
**Technical quality of care**	** **	** **	** **	** **	** **	** **
Mean	11.09	10.64	10.86	14.2	8.2	11.2
(SD)	(4.24)	(4.32)	(4.29)	(3.22)	(3.17)	(4.38)
**Delivery complications**	** **	** **	** **	** **	** **	** **
No	(30.9%)	(85.6%)	(58.2%)	(94.7%)	(81.7%)	(88.2%)
Yes	(69.1%)	(14.4%)	(41.8%)	(5.0%)	(18.3%)	(11.7%)
Don't know	(0.0%)	(0.0%)	(0.0%)	(0.3%)	(0.0%)	(0.2%)
**Hours after delivery (after_del_hours)**	** **	** **	** **	** **	** **	** **
Mean	5.49	5.66	5.58	4.63	4.47	4.55
(SD)	(4.86)	(4.99)	(4.92)	(3.37)	(3.69)	(3.53)
**Frequency of doctor visits**	** **	** **	** **	** **	** **	** **
0	(65.3%)	(81.4%)	(73.3%)	(20.7%)	(97.0%)	(58.8%)
1 time a day	(26.0%)	(18.2%)	(22.1%)	(47.0%)	(2.7%)	(24.8%)
2 times a day	(7.0%)	(0.4%)	(3.7%)	(17.3%)	(0.3%)	(8.8%)
3 times a day	(1.8%)	(0.0%)	(0.9%)	(8.7%)	(0.0%)	(4.3%)
4 times a day	(0.0%)	(0.0%)	(0.0%)	(6.3%)	(0.0%)	(3.2%)
**Frequency of nurse visits**	** **	** **	** **	** **	** **	** **
0	(27.4%)	(21.4%)	(24.4%)	(0.3%)	(60.7%)	(30.5%)
1 time a day	(34.0%)	(36.8%)	(35.4%)	(0.7%)	(30.3%)	(15.5%)
2 times a day	(22.5%)	(23.5%)	(23.0%)	(8.0%)	(8.0%)	(8.0%)
3 times a day	(13.3%)	(11.2%)	(12.3%)	(34.7%)	(1.0%)	(17.8%)
More than 3 times	(2.8%)	(7.0%)	(4.9%)	(56.3%)	(0.0%)	(28.2%)
**Family planning**	** **	** **	** **	** **	** **	** **
Already using one	(0.0%)	(0.0%)	(0.0%)	(2.0%)	(13.8%)	(7.9%)
Planning to use	(10.2%)	(22.1%)	(16.1%)	(22.0%)	(27.2%)	(24.6%)
Not planning to use	(88.8%)	(71.2%)	(80.0%)	(75.7%)	(50.7%)	(63.2%)
Don't know	(1.1%)	(6.7%)	(3.9%)	(0.3%)	(8.4%)	(4.3%)
**Cesarean section**	** **	** **	** **	** **	** **	** **
No	(97.9%)	(99.3%)	(98.6%)	(100.0%)	(99.3%)	(99.7%)
Yes	(2.1%)	(0.7%)	(1.4%)	(0.0%)	(0.7%)	(0.3%)
**Wait time for the first examination**	** **	** **	** **	** **	** **	** **
Less than 10 minutes	(89.8%)	(25.6%)	(57.7%)	(68.3%)	(54.3%)	(61.3%)
10–19 minutes	(6.7%)	(68.1%)	(37.4%)	(21.0%)	(41.0%)	(31.0%)
20 minutes or more	(3.5%)	(6.3%)	(4.9%)	(10.7%)	(4.7%)	(7.7%)
**Time in facility before delivery**	** **	** **	** **	** **	** **	** **
Less than 1 hour	(32.3%)	(26.7%)	(29.5%)	(29.3%)	(30.3%)	(29.8%)
More than 1 hour	(67.7%)	(73.3%)	(70.5%)	(70.7%)	(69.7%)	(70.2%)

## Discussion

Most significantly, this small-scale intervention study has demonstrated the potential for a QI intervention in Uttar Pradesh to achieve significant improvements in women’s childbirth experiences in public facilities. In the intervention sites the improvements were driven by change strategies designed by frontline staff and tailored to the context and capacity of the facility in which they were tested. No change strategies required financial inputs or changes to infrastructure at the facility. The improvements occurred in both overall PCMC, and within each of the three sub domains. Although change strategies were developed around specific PCMC indicators (i.e. provider explains the purpose of tests and medicines to the patient), the impact of this improvement work as reported by women who participated in the endline survey was much broader, affecting respondents’ overall experiences of respect, dignity, empowerment, and their trust in their providers.

Additionally, this study found that the intervention made improvements in outcomes beyond women’s experiences of care. For example, intervention facilities were associated with reported decreases in delivery complications and additional visits from a doctor and nurses. Potential explanations for this include overall leadership from the Government of India in investing in quality of care initiatives aimed to improve both clinical and experiential care, such as the Labor Room Quality Improvement Initiative (LaQshya) and support for the PCMC improvement work from the National Health Mission (NHM) of Uttar Pradesh. More directly, attention and external support for better quality may have increased the enthusiasm and empowered nurses, dais, and other staff to address aspects of quality beyond just those targeted in the intervention.

It should also be noted that intervention facilities had previously participated in a major quality of care initiative focused on improving clinical quality of care through use of a validated childbirth checklist [27]. While that intervention ultimately did not assure clinical quality standards, it did improve many processes and it is plausible that these facilities were primed to improve clinical quality of care and person-centered maternity care and may not reflect the ability of other public facilities in Uttar Pradesh to take up this type of intervention. Counter to our findings on improvements in clinical quality and women’s experiences of care, patients also reported longer wait times in intervention vs. control facilities. This is in line with our findings that intervention facilities also reported more frequent visits from nurses and doctors. That is, women may be reporting longer wait times due to more time and attention paid for each patient. While wait times may be longer, findings also suggest an improvement in women’s experience of care and clinical quality of care.

This intervention study confirms the findings of other studies that indicate lower-level staff provide more attentive, respectful, and person-centered maternity care than doctors and nurses, and that the relative importance of these staff in smaller facilities may be what makes them more patient friendly [19, 28, 29]. This study also confirms evidence that better PCMC is positively associated with better clinical care [10]. Clinical and experiential quality are interlinked, with improvements in one area synergistic to improvements in the other.

This study has a number of limitations. First, the intervention was implemented in a small number of facilities (3), which makes a clustering effect likely. Additionally, it is possible that our results are reflective of a Hawthorne effect; PCMC behaviors may have changed due to an awareness among staff that their behaviors would be measured via the patient survey. Furthermore, it is possible that improvements in the overall and PCMC subdomain scores may be partially attributed to a halo effect, as sensitization to PCMC overall could have contributed to increased awareness and behavior change among staff as a whole. Response bias among women who delivered may have effected their answers, or anxiety about treatment by providers may have made women unwilling to accurately describe negative experiences, and while survey teams were not told which sites were intervention or controls, they could have seen posters or other indications allowing them to determine this and so bias the way in which they conducted their surveys. While we selected facilities based on an expectation that the BetterBirth study would assure a baseline of clinical quality, that may not have happened [27]. Externally, government initiatives and support may have positively impacted the success of the intervention. The LaQshya initiative was rolled out during the time that our intervention was being implemented, thus highlighting a national commitment to improving quality of maternity care for facility staff. Finally, as the study was a collaboration involved foreign researchers and an Indian government agency, the NHM, district or facility leadership may have responded to ideas developed as part of the QI intervention with more enthusiasm than would be the case in a nationally rolled out initiative.

## Conclusion

As India seeks to identify strategies to improve the way that services are provided for women giving birth in government facilities, this study provided strong data that improvement is possible. The QI methodology applied in Uttar Pradesh has led to improvements in women’s experiences, and merits replication and testing to optimize how it can best be implemented at a larger scale. It moreover offers a new, standardized, and replicable model to make that positive change take place. This study shows what is possible: whether it can be expanded to all facilities and all populations remains unknown. We can imagine that lower clinical quality in facilities which were not part of prior quality improvement studies, which are farther from the capitol city of Lucknow, or which have worse management may distract or demoralize staff, making the team-based improvements which were the core of this intervention more difficult and less effective. An additional study is planned to assess whether the findings of this study can be replicated in sites not previously involved in quality improvement, with less intensive support from an external QI specialist, and to study how long the effects of the intervention can be sustained once external technical assistance ceases.
